# iNOS-derived nitric oxide promotes glycolysis by inducing pyruvate kinase M2 nuclear translocation in ovarian cancer

**DOI:** 10.18632/oncotarget.16523

**Published:** 2017-03-23

**Authors:** Linlin Li, Lingqun Zhu, Bingtao Hao, Wenwen Gao, Qianli Wang, Keyi Li, Meng Wang, Mengqiu Huang, Zhengjun Liu, Qiaohong Yang, Xiqing Li, Zhuo Zhong, Wenhua Huang, Guanghui Xiao, Yang Xu, Kaitai Yao, Qiuzhen Liu

**Affiliations:** ^1^ Cancer Research Institute, Southern Medical University, Guangzhou 510515, China; ^2^ Guangdong Provincial Key Laboratory of Cancer Immunotherapy Research, Guangzhou 510515, China; ^3^ Guangzhou Key Laboratory of Tumor Immunology Research, Southern Medical University, Guangzhou 510515, China; ^4^ Department of Vascular Surgery, Nanfang Hospital, Southern Medical University, Guangzhou 510515, China; ^5^ School of Basic Medical Sciences, Guangzhou University of Chinese Medicine, Guangzhou 510006, China; ^6^ Department of Pathology, Yale University School of Medicine, New Haven, CT 06520, USA; ^7^ Department of Oncology, Henan Provincial People's Hospital, Zhengzhou 450003, Henan, China; ^8^ Department of Oncology, Guangzhou Hospital of Integrated Traditional and Western Medicine, Guangzhou 510800, China; ^9^ Department of Human Anatomy, Southern Medical University, Guangzhou 510515, China

**Keywords:** nitric oxide, iNOS, Warburg effect, PKM2, EGFR/ERK2

## Abstract

Aerobic glycolysis is essential for tumor growth and survival. Activation of multiple carcinogenic signals contributes to metabolism reprogramming during malignant transformation of cancer. Recently nitric oxide has been noted to promote glycolysis but the mechanism remains elusive. We report here the dual role of nitric oxide in glycolysis: low/physiological nitric oxide (≤ 100 nM) promotes glycolysis for ATP production, oxidative defense and cell proliferation of ovary cancer cells, whereas excess nitric oxide (≥ 500 nM) inhibits it. Nitric oxide has a positive effect on glycolysis by inducing PKM2 nuclear translocation in an EGFR/ERK2 signaling-dependent manner. Moreover, iNOS induced by mild inflammatory stimulation increased glycolysis and cell proliferation by producing low doses of nitric oxide, while hyper inflammation induced iNOS inhibited it by producing excess nitric oxide. Finally, iNOS expression is abnormally increased in ovarian cancer tissues and is correlated with PKM2 expression. Overexpression of iNOS is associated with aggressive phenotype and poor survival outcome in ovarian cancer patients. Our study indicated that iNOS/NO play a dual role of in tumor glycolysis and progression, and established a bridge between iNOS/NO signaling pathway and EGFR/ERK2/PKM2 signaling pathway, suggesting that interfering glycolysis by targeting the iNOS/NO/PKM2 axis may be a valuable new therapeutic approach of treating ovarian cancer.

## INTRODUCTION

One of fundamental properties of cancer cells is that they underwent metabolic reprogramming from oxidative phosphorylation to aerobic glycolysis during carcinogenesis [1]. Cancer cells rely primarily on aerobic glycolysis to sustain their proliferation, this phenomenon is known as “Warburg effect” [2]. Although aerobic glycolysis is inefficient for ATP generation as compared to oxidative phosphorylation, it is optimized to meet the need for cell proliferation by stimulation of the carbon flux to biosynthesis pathways, increase of NADPH for anabolism and oxidative defense [3]. Enhanced glycolysis is achieved by accumulation or mutation of transcription factors (HIF-1α, MYC, and P53) [4], higher rate of glucose uptaken, increased expression of glycolytic enzymes and attenuated oxidative phosphorylation (OXPHOS) [3]. However, the mechanism of metabolic rewiring in cancer cells remains to be an open question.

Nitric oxide (NO) is a highly reactive free radical which is synthesized from l-arginine and oxygen by nitric oxide synthase (NOS) in cells [5]. Nitric oxide functions as a signal transducer regulating multiple physiological and pathological processes including blood flow regulation, smooth muscle relaxation, neurotransmission and cancer [6]. There are three NOS isoforms in human cells [7]. Neuronal NOS (nNOS) and endothelial NOS (eNOS) are constitutively expressed in cells and produce nanomolar concentrations of nitric oxide in calcium (Ca^2+^)-dependent way. Unlike the two isoforms, inducible NOS (iNOS) expression is induced by inflammatory cytokines, hypoxia and oxidative stress in Ca^2+^-independent way and generates micromolar concentrations of nitric oxide [8–10]. It has been noted that NOSs/NO have biphasic effects in carcinogenesis, progression and therapeutics of tumors dependent on the concentration and duration of nitric oxide exposure, NOS isoforms and micro-environmental conditions [6].

Nitric oxide has been well established as a modulator of cellular bioenergetics and has aroused people's interest on its effects on glycolysis of tumors including ovarian cancer, melanoma and breast cancer [11, 12]. The effect of nitric oxide on glycolysis is complicated and appears to be context dependent [13]: nitric oxide promotes glycolysis by sustaining HIF-1α protein stability in oral squamous cell carcinoma [14] while, under inflammatory state, high dose nitric oxide derived from iNOS inhibits glycolysis by S-ntirosylation of glyceraldehyde-3-phosphate dehydrogenase (GAPDH) [15]. Nitric oxide also stimulates glycolysis in astrocytes without effect on that in neurons [13]. So the interesting question is whether nitric oxide plays a biphasic role in cancer glycolysis [12].

Pyruvate kinase (PK) is a rate-limiting enzyme in the last step of glycolysis pathway which catalyzes the pyruvate production from phosphoenolpyruvate [16]. Among four isoforms of PK (PKL, PKR, PKM1 and PKM2), PKM2 is the predominant isoform in tumor cells and exerts an important role in glycolysis and tumor malignancy [17]. PKM2 exists in monomer, dimer and tetramer forms. Cytosolic PKM2 exists as a tetrameric form with a high activity of pyruvate kinase [18]. Under various stimuli such as oxidative stress and growth factor, the tetramer of PKM2 is converted to dimer and translocates into nucleus to function as a protein kinase [19, 20]. It phosphorylates and activates the transcription factors like HIF-1α and MYC for glycolytic gene expression [21]. It remains unclear whether PKM2 is regulated by NO signaling.

Epithelial ovarian cancer is the fifth most common cause of cancer death in women and the leading cause of death among gynaecological malignancies [22, 23]. We herein show a dual role of NO/iNOS in glycolysis of ovarian cancer cells: low/physiological nitric oxide promotes glycolysis while excessive nitric oxide inhibits it. We further demonstrate that iNOS produced-nitric oxide promotes glycolysis by inducing the PKM2 nuclear translocation in EGFR/ERK2-dependent manner. Moreover, the higher expression of iNOS in ovarian cancer specimens can predict poor prognosis, supporting the idea of the iNOS as a helpful prognostic marker and a potential therapeutic target for ovary cancer.

## RESULTS

### Dual effects of nitric oxide on glycolysis in ovarian cancer cell

**Figure 1:**
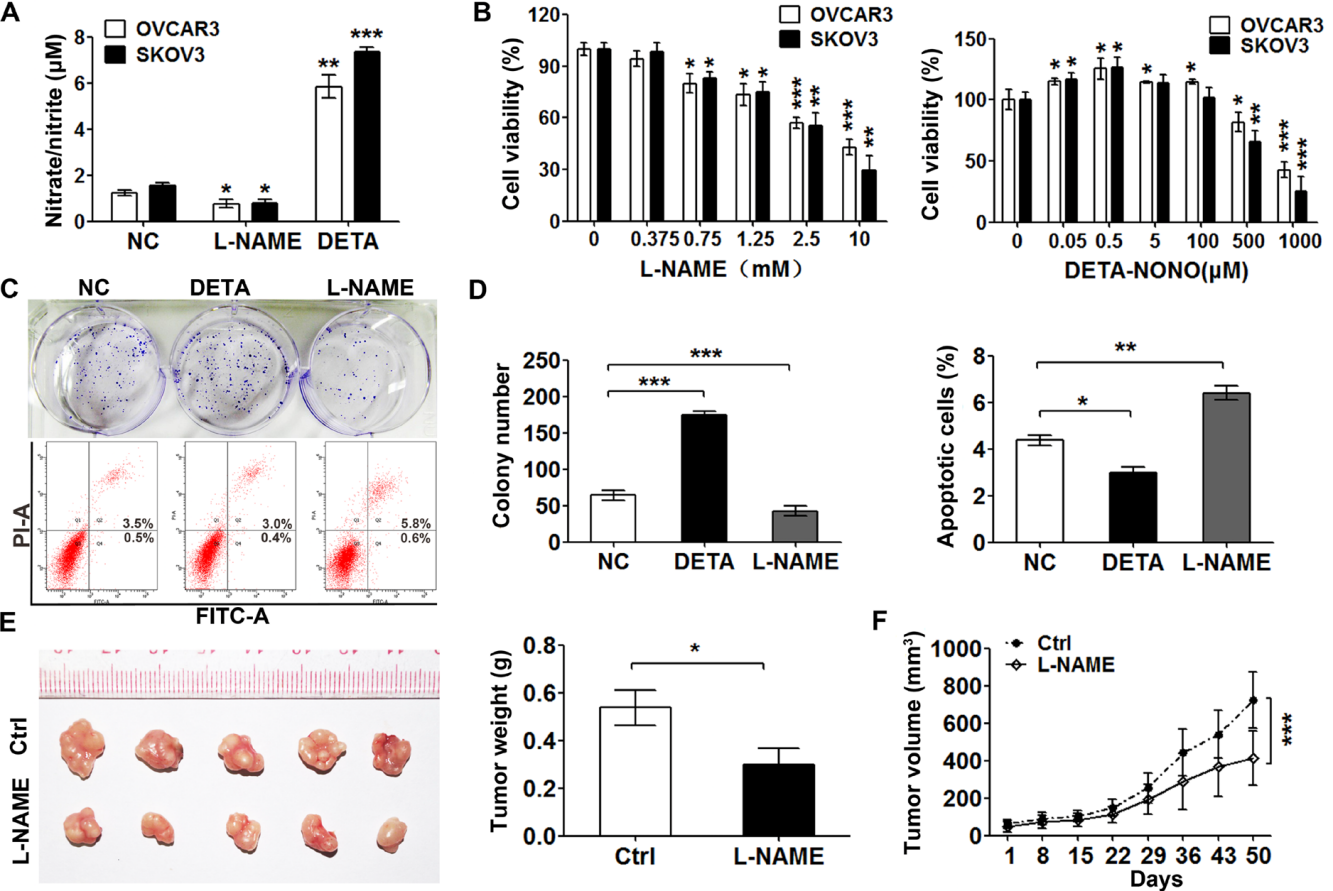
Low level of exogenous nitric oxide promotes cell proliferation (**A**) DETA-NONOate increased the nitric oxide production and L-NAME decreased it. Nitrate/nitrite concentration was measured in the media of OVCAR3 and SKOV3 cells by Griess assay after treatment with DETA-NONOate (50 μM) or L-NAME (1 mM) for 24 hours. (*n* = 3, one-way ANOVA). (**B**) Effects of L-NAME and DETA-NONOate on the cell viability of SKOV3 and OVCAR3. Cells treated with gradient concentrations of L-NAME (left panel) or DETA-NONOate (right panel) for 48 hours were subjected to CCK8 assay (*n* =3, one-way ANOVA). (**C**, **D**) Nitric oxide enhanced the colony formation and anti-apoptosis abilities of SKOV3 cell. Cells treated with DETA-NONOate (50 μM) or L-NAME (1 mM) were subjected to colony formation assay (upper panel) and cell apoptosis assay (lower panel) (*n* =3, One-way ANOVA). (**E**, **F**) Inhibition of nitric oxide decreased ovarian cancer growth *in vivo*. Subcutaneous xenografts of SKOV3 cells in nude mice were treated with L-NAME (50mg/kg) or 0.9% NaCl (Ctrl). Tumor weights (mg) were determined after the treatment was completed at day 50 (*n* =5, Student's *t* test) (E). F. Tumor volume (mm^3^) was measured at the indicated treatment days 8, 15, 22, 29, 36, 43 and 50. Data represent the mean ± s.e.m (error bars); *p < 0.05; **p < 0.01; ***p < 0.001.

To evaluate the effects of nitric oxide in ovarian cancer cells, the level of nitric oxide was measured by detecting nitrosating species in the media of the OVCAR3 and SKOV3 cells after treatment with NOS inhibitor L-NAME or NO donor DETA-NONOate by Griess assay. The amount of nitrosating species in the OVCAR3 and SKOV3 cells were higher than that treated with L-NAME and one fifth of that released by 50 μM DETA-NONOate (Figure 1A). Moreover, the basal level of nitric oxide in the OVCAR3 and SKOV3 cells is about 10 nM, since DETA-NONOate of 50 μM produced a steady-state nitric oxide of approximately 50 nM [24]. The cell viabilities of OVCAR3 and SKOV3 cells were detected by CCK8 assay after 48-hour of treatment with NO donor or L-NAME at gradient concentrations. The results showed that NOS inhibitor reduced the survival of the cells in a dose-dependent manner (Figure 1B, left panel). We observed that low dose of DETA-NONOate promoted the cell proliferation while the cells died after the treatment with high concentration of NO donor (Figure 1B, right panel). The colony formation assay gave a similar result (Figure 1C, upper panel and Figure 1D, left panel). Consistently, supplying nitric oxide with DETA-NONOate slightly decreased the percent of total apoptosis cells, while L-NAME treatment increased apoptosis around 1.6-fold comparing with the untreated cells (Figure 1C, lower panel and Figure 1D, right panel). The xenograft experiment with SKOV3 cells showed that tumors treated with L-NAME grew significantly slower than the control groups did (Figure 1E–1F). Taken together, these results indicate that low dose of nitric oxide supplying promotes cancer cell growth while high concentration kills cells.

**Figure 2:**
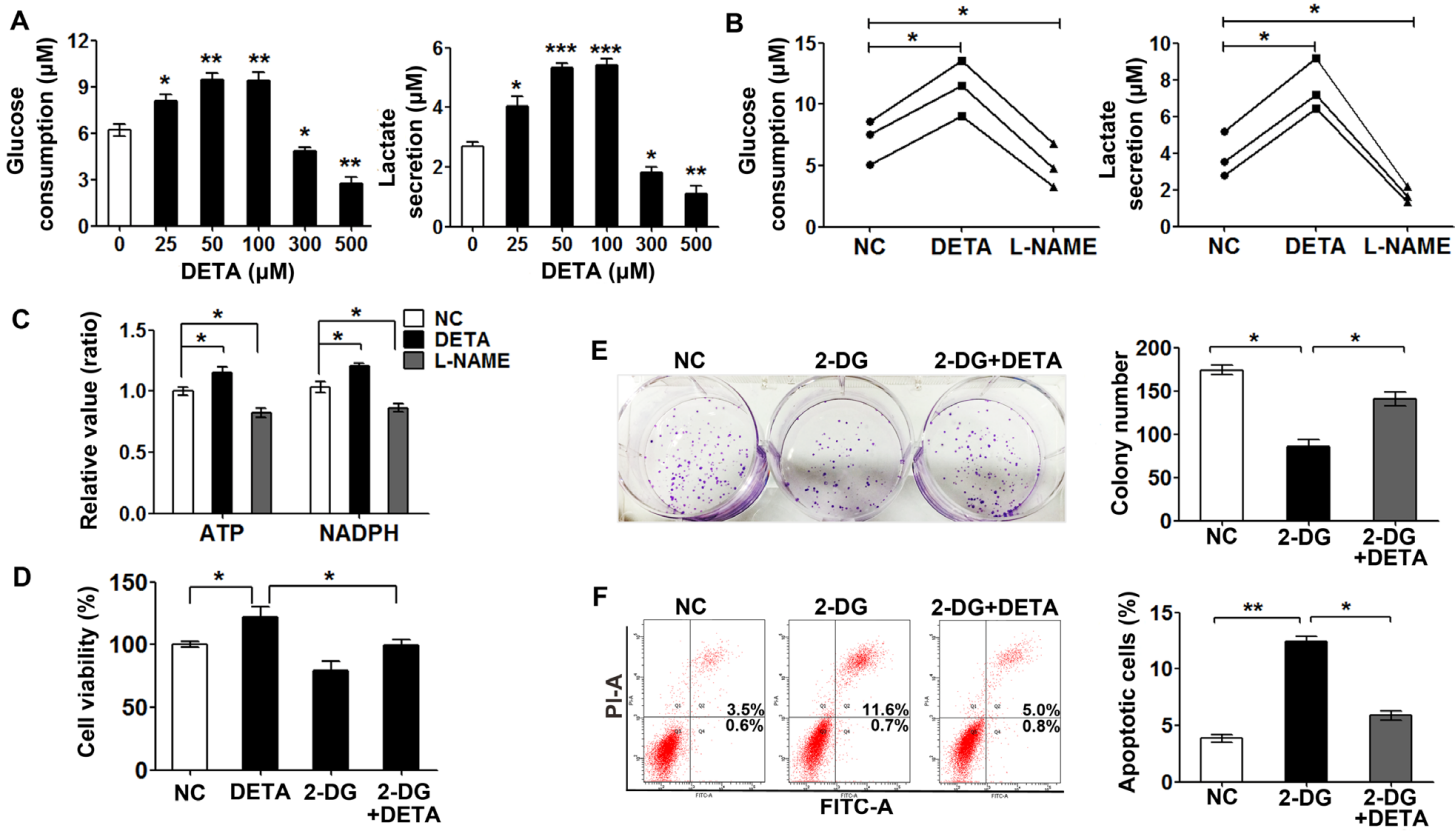
Dual role of exogenous nitric oxide in glycolysis (**A**) Low doses of nitric oxide promoted glycolysis and excess nitric oxide inhibited it in SKOV3 cells as measured by glucose consumption and lactate secretion assay. Cells were treated with different concentrations of DETA-NONOate for 24 hours. (**B**) Nitric oxide promoted glycolysis as measured by glucose consumption and lactate secretion assay in SKOV3, OVCAR3 and ES-2 cells. Cells were treated with L-NAME (1 mM) or DETA-NONOate (50 μM) for 48 hours. (**C**) Fold changes of ATP production and NADPH generation in SKOV3 cells treated with L-NAME (1 mM) or DETA-NONOate (50 μM) for 48 hours. (**D**) 2-DG reversed nitric oxide-induced cell proliferation. SKOV3 cells treated with DETA-NONOate (50 μM) and (or) 2-DG (1 mM) for 48 h were subjected to CCK8 assay. (**E**) SKOV3 cells were plated in low density in 6-well plate, and then treated with DETA-NONOate (50 μM) and 2-DG (1 mM) for 14 days, colonies were stained with crystal violet. (**F**) SKOV3 cells treated with DETA-NONOate (50 μM) and (or) 2-DG (1 mM) for 48 h were subjected to flow cytometric analysis with propidium iodide and Annexin V staining. All data represent the mean ± s.e.m (error bars); *n* ≥ 3; *p < 0.05; **p < 0.01; ***p < 0.001.

To investigate the effects of nitric oxide on glycolysis, we detected the glucose consumption and lactate secretion in SKOV3 cells after exposure to DETA-NONOate. Glucose consumption and lactate secretion of the cells treated with DETA-NONOate (≤ 100 μM) were increased in a dose-dependent manner while that with high concentrations (≥ 300 μM) reduced (Figure 2A). Consistent with the effect of low dose NO donor treatment, inhibition of NOSs decreased the glucose consumption and lactate secretion in the SKOV3, OVCAR3 and ES-2 cells (Figure 2B). The main products of glucose metabolism are adenosine triphosphate (ATP), nicotinamide adenine dinucleotide phosphate (NADPH) and precursors of macromolecule biosynthesis, which are critical for cancer cell addressing energy needing, oxidative stress and proliferation requirement [25, 26]. So we detected the production of ATP and NADPH in cells treated with low dose DETA-NONOate and L-NAME. The result clearly showed that the supply of nitric oxide significantly increased NADPH and ATP production and *vice versa* (Figure 2C). These results suggested that nitric oxide promotes glycolysis in cancer cells to coordinate energy generation, biosynthesis and oxidative defense for their unrestricted growth.

To answer whether glycolysis is involved in the enhanced cancer growth with a low dose of nitric oxide supply, we inhibited glycolysis with a competitive inhibitor 2-dexxyglucose (2-DG). We observed that the effects of nitric oxide on cell viability, colony formation and anti-apoptosis were attenuated by 2-DG (Figure 2D–2F), suggesting that the effects of exogenous nitric oxide on cell proliferation and anti-apoptosis property depends, at least in part, on glycolysis. Taken together, these results indicated that the dual role of nitric oxide on glycolysis and cell proliferation is concentration dependent, low/physiolocal level of nitric oxide in cancer cells play a critical role in glycolysis and cell proliferation, and inhibition of nitric oxide production impaired cancer cell survival.

### Nitric oxide induces PKM2 nuclear translocation and promotes glycolytic genes expression

**Figure 3:**
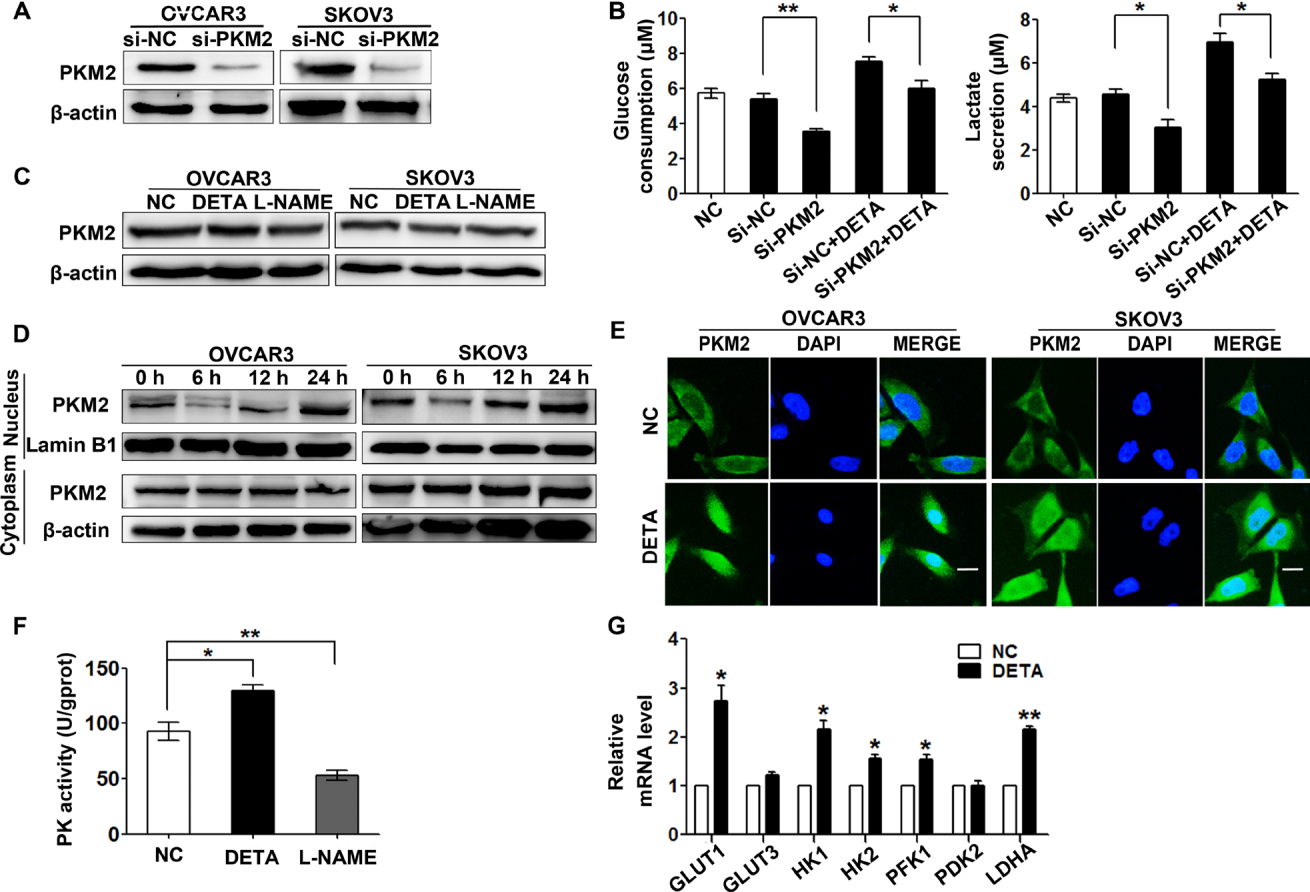
Nitric oxide induces PKM2 nuclear translocation (**A**) PKM2 was knocked down in OVCAR3 and SKOV3 cells by PKM2 siRNA as determined by immunoblotting assay. (**B**) Nitric oxide-induced glycolysis was partially reversed by si-PKM2. SKOV3 cells treated with si-NC, si-PKM2, DETA-NONOate or their combination for 48 hours were subjected to glucose consumption and lactate secretion detection. (**C**) Nitric oxide did not change PKM2 expression. SKOV3 cells treated with DETA-NONOate or L-NAME for 48 hours were subjected to immunoblotting assay. (**D**) Nitric oxide induced PKM2 nuclear accumulation as analyzed by immunoblotting. Nuclear and cytoplasmic proteins were extracted from OVCAR3 and SKOV3 cells after treated with DETA-NONOate for indicated hours. (**E**) The subcellular localization of PKM2 was visualized by immunofluorescence staining in OVCAR3 and SKOV3 cells after treated with DETA-NONOate for 24 hours. Nuclei were stained with DAPI (blue), original magnification: 400× and scale bar represents 20 μm. (**F**) Nitric oxide increased pyruvate kinase activity. SKOV3 cells treated with DETA-NONOate or L-NAME for 24 hours were subjected to PK activity detection. (**G**) Nitric oxide increased mRNA expression of glycolytic genes of *GLUT1*, *GLUT3*, *HK1*, *HK2*, *PFK1*, *PDK2*, and *LDHA*. SKOV3 cells treated with DETA-NONOate for 24 hours were subjected to real-time PCR assay. Columns, mean (*n* = 3); bars, s.e.m; *p < 0.05; **p < 0.01 in B, F and G. Cytoplasmic β-actin and nuclear Lamin B1 were loaded as internal reference for cytoplasmic and nuclear extractions respectively in A, C and D.

PKM2 is a critical rate-limiting enzyme in glycolysis and highly expressed in ovarian cancer cells [27]. To test whether PKM2 is involved in the regulation of glycolysis by nitric oxide in ovarian cancer cells, we knocked down PKM2 with siRNA to detect glucose consumption and lactate secretion. The results showed that knockdown of PKM2 reversed the NO donor induced glycolysis in SKOV3 cells (Figure 3A–3B). We also found that supplying nitric oxide with DETA-NONOate or inhibition of NOS by L-NAME didn't change PMK2 expression by immunoblotting assay (Figure 3C). Previous studies have showed that nuclear PKM2 mediates cell proliferation and metabolic reprogramming in cancer cells [28]. We observed the time-depended accumulation of PKM2 protein in the nuclear after NO donor treatment, while the cytoplasmic PKM2 remained at the same level (Figure 3D). We also examined PKM2 nuclear translocation by immunofluorescence staining and found that PKM2 was accumulated in nucleus after 24-hour DETA-NONOate treatment (Figure 3E).

It was reported that nuclear PKM2 promotes the transcription of glycolytic genes [21]. Using the real-time PCR assay to detect the expressions of the glycolytic genes, the data showed that the mRNA level of the glucose transporter genes (*GLUT1*, *GLUT3*), glycolytic enzymes hexokinase genes (*HK1*, *HK2*), phosphofructokinase 1 gene (*PFK1*), 3-phosphoinositide-dependent protein kinase 2 gene (*PDK2*) and lactate dehydrogenase gene (*LDH*) were up-regulated in SKOV3 cells treated with NO donor (Figure 3G). These results indicated that exogenous nitric oxide induces PKM2 nuclear translocation and promotes glycolysis in ovarian cancer cells. In addition, the provision of nitric oxide with DETA-NONOate increased the pyruvate kinase activity and inhibition of NOSs reduced its activity (Figure 3F).

### Nitric oxide-induced PKM2 nuclear translocation is EGFR/ERK2-signaling dependent

**Figure 4:**
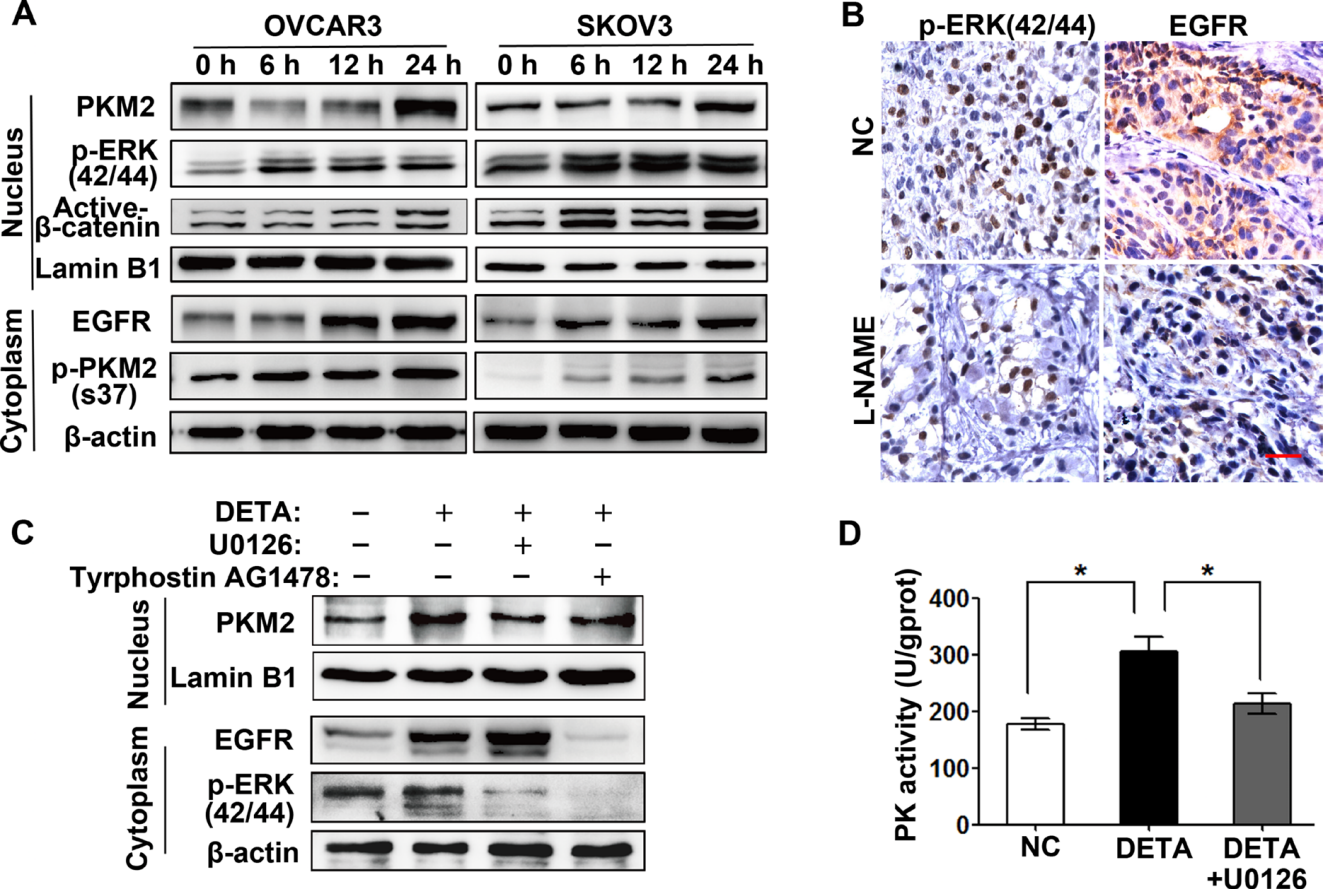
Nitric oxide-induced PKM2 nuclear translocation is EGFR/ERK2-signaling dependent (**A**) Nitric oxide activates EGFR/ERK2/PKM2 signaling. SKOV3 and OVCAR3 cells treated with DETA-NONOate for 6, 12 or 24 hours were subjected to immunoblotting assay with indicated antibodies. (**B**) Nitric oxide inhibition reduced the EGFR expression and ERK2 phosphorylation *in vivo*. Immunohistochemistry staining of the primary tumor tissues was performed with indicated antibodies. Original magnification: 400× and scale bar represents 20 μm. (**C**) Inhibition of EGFR/ERK2-singaling abrogated the nitric oxide-induced PKM2 nuclear translocation. SKOV3 cells treated with U0126 (20 μM), Tyrphostin AG1478 (100 nM), DETA-NONOate or their combination for 24 hours were subjected to immunoblotting. (**D**) ERK2 inhibition partially abrogated the nitric oxide-enhanced pyruvate kinase activity. PK activity was detected in SKOV3 cells after treated with DETA-NONOate or U0126. Columns, mean (*n* = 3); bars, s.e.m; *p < 0.05.

It's reported that epidermal growth factor receptor (EGFR)-activated extracellular signal-regulated kinase (ERK2) promotes the nuclear translocation of PKM2 by directly binding PKM2 and phosphorylating it at Ser37 residue [19]. We want to know whether the PKM2 nuclear translocation induced by nitric oxide is mediated by ERK2. Figure 4A showed that low dose of NO donor increased the protein level expression of EGFR, the phosphorylation level of ERK2 (Thr185/Tyr187) and PKM2 (S37) in OVCAR3 and SKOV3 cells. The NOS inhibitor treatment also reduced EGFR expression and ERK2 phosphorylation level in the tumor tissues of xenograft mouse by using immunohistochemistry assay (Figure 4B). The previous study reported that nuclear PKM2 interacts with and activates β-catenin as a transcription cofactor to regulate glycolytic genes [19]. We examined the non-phosphorylated (“activated”) form of β-catenin in nucleus and found the active form of β-catenin increased upon NO donor exposure in OVCAR3 and SKOV3 cells (Figure 4A). Moreover, NO donor induced nuclear translocation of PKM2 was abrogated by MEK/ERK inhibitor (U0126) and EGFR inhibitor (AG1478) (Figure 4C). The pyruvate kinase activity was also attenuated by MEK/ERK inhibitor U0126 (Figure 4D). Collectively, nitric oxide induced PKM2 nuclear translocation is dependent on EGFR/ERK2 signaling pathway.

Previous studies reported that nitric oxide regulates tumor biology through activating the soluble guanylate cyclase (sGC) to generate the secondary messenger cyclic GMP (cGMP) [29]. To address whether sGC/cGMP signaling is involved in nitric oxide induced-glycolysis, we blocked cGMP signaling in SKOV3 cells by treating with guanylate cyclase inhibitor ODQ (1H-[1,2,4] Oxadiazolo [4,3-a]quinoxalin-1-one) [30]. Treatment with ODQ slightly attenuated the positive effects of nitric oxide on glucose consumption and lactated secretion (Supplementary Figure 1A), which indicated that the nitric oxide-stimulated glycolysis in ovarian cancer cells might be also mediated by sGC-cGMP signaling.

Another important way of nitric oxide signaling is posttranslational modification of S-nitrosylation on specific cysteine residues in proteins [31]. To explore the role of S-nitrosylation in nitric oxide-induced glycolysis, we analyzed the S-nitrosylation of PKM2 by “biotin-switch” method. Results showed that exogenous supplying nitric oxide with DETA-NONOate had little effect on the S-nitrosylation level of PKM2. It surprised us that non-specific NOS inhibitor L-NAME markedly reduced the S-nitrosylated PKM2 (Supplementary Figure 1B). These suggests that the role of exogenous NO on PKM2 did not mediated by S-nitrosylation modification of PKM2, but endogenous NO formed by three NOS isoforms might regulate PKM2 S-nitrosylation.

Nitric oxide can activate protein kinase B (AKT) and signal transducer and activator of transcription 3 (STAT3), which play key roles in multiple cellular processes including tumor glucose metabolism [32, 33]. To investigated whether they were involved in NO-mediated glycolysis. We performed immunoblotting assay and didn't observe the phosphorylation level change of both AKT and STAT3 in ovarian cancer cells upon treatment with NO donor or NOS inhibitor (Supplementary Figure 1C). This result is inconsistent with published data [32], which might due to different concentration of NO donor used in the experiment.

### Gene expression profiling analysis of NOSs isoforms in clinical ovary cancer tissues

**Figure 5:**
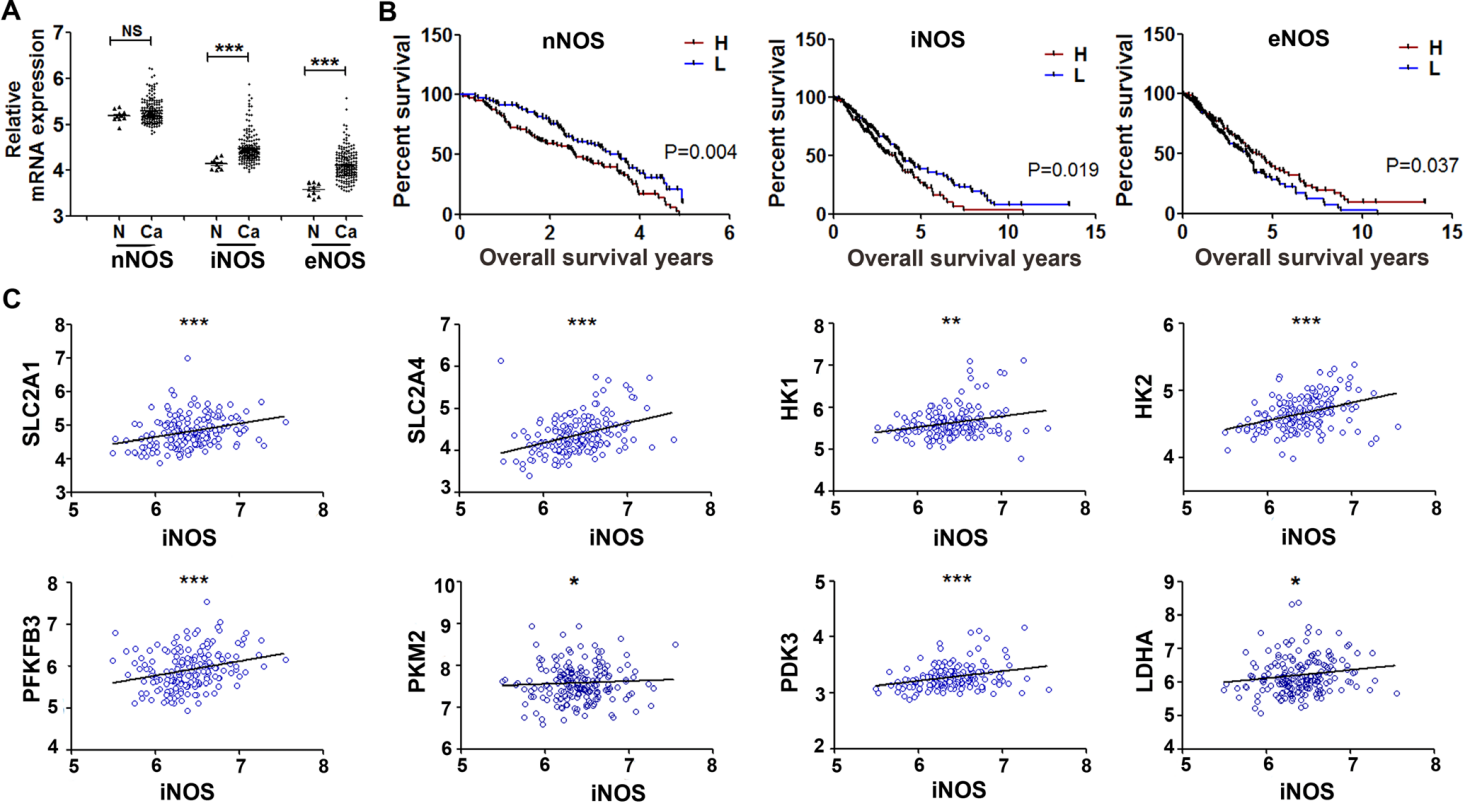
Gene expression profiling analysis of NOS isoforms in clinical ovary cancer tissues Global gene expression profiling downloaded from Gene Expression Omnibus (GEO, GSE26712 and GSE14764) contains 250 ovarian cancer samples and 10 normal ovarian epithelial samples. (**A**) Scatter plot showing the comparison of nNOS, iNOS, eNOS in ovarian cancer samples and normal ovarian epithelial samples. (**B**) Effects of nNOS, iNOS and eNOS on overall survival time of patients was analyzed by Wilcoxon test of Kaplan-Meier. (**C**) Scatter plot showing the positive correlation of iNOS mRNA values (x axis) with glycolytic genes *SLC2A1*, *SLC2A4*, *HK1*, *HK2*, *PFKFB3*, *PKM2*, *PDK3*, and *LDHA* (y axis) in ovarian cancer patients. *P < 0.05; **P < 0.01; ***P < 0.001 in A and C.

The above data indicate that nitric oxide promotes glycolysis of cancer cells and tumor growth through inducing PKM2 nuclear translocation in EGFR/ERK2-dependent manner. There are three NOS isoforms in human cells, to interrogate which NOS isoform correlate with glycolysis and progression in ovarian cancer, we exploited the global gene expression profiling from Gene Expression Omnibus (GEO, GSE26712 and GSE14764). The mRNA expressions of iNOS and eNOS were significantly higher in 250 primary ovarian tumors as compared to the 10 normal ovarian epithelial tissues (Figure 5A). Kaplan–Meier analysis of the overall survival in ovarian cancer patients showed that the patients with higher iNOS or nNOS level experienced significantly shorter overall survival than the patients in the lower iNOS or nNOS level. In contrast, the patient with higher eNOS level exhibited optimistic survival than the patients with lower eNOS (Figure 5B).

Among the three NOS isoforms, iNOS stands apart as it is stimulated by inflammatory cytokines, lipopolysaccharide endotoxin or oxidative stress and generating more nitric oxide than the constitutive nNOS and eNOS [10]. Next, we analyzed the correlation between iNOS mRNA expression and glycolytic genes. iNOS was positively correlated with expression of *PKM2* and most of other glycolytic genes including *SLC2A1* (*GLUT1*), *SLC2A4* (*GLUT4*), *HK1*, *HK2*, *PFKFB3*, *PDK3*, and *LDHA* (Figure 5C). On the contrary to iNOS, the expression of eNOS mRNA was negatively correlated with most glycolytic genes including *SLC2A1*, *SLC2A4*, *HK1*, *HK2*, *PFKFB3*, *PDK3*, and *LDHA* (Supplementary Figure 2A). These results indicated that iNOS might play a role for ovarian cancer progression through glycolysis.

### iNOS contributes to nitric oxide-mediated glycolysis

**Figure 6:**
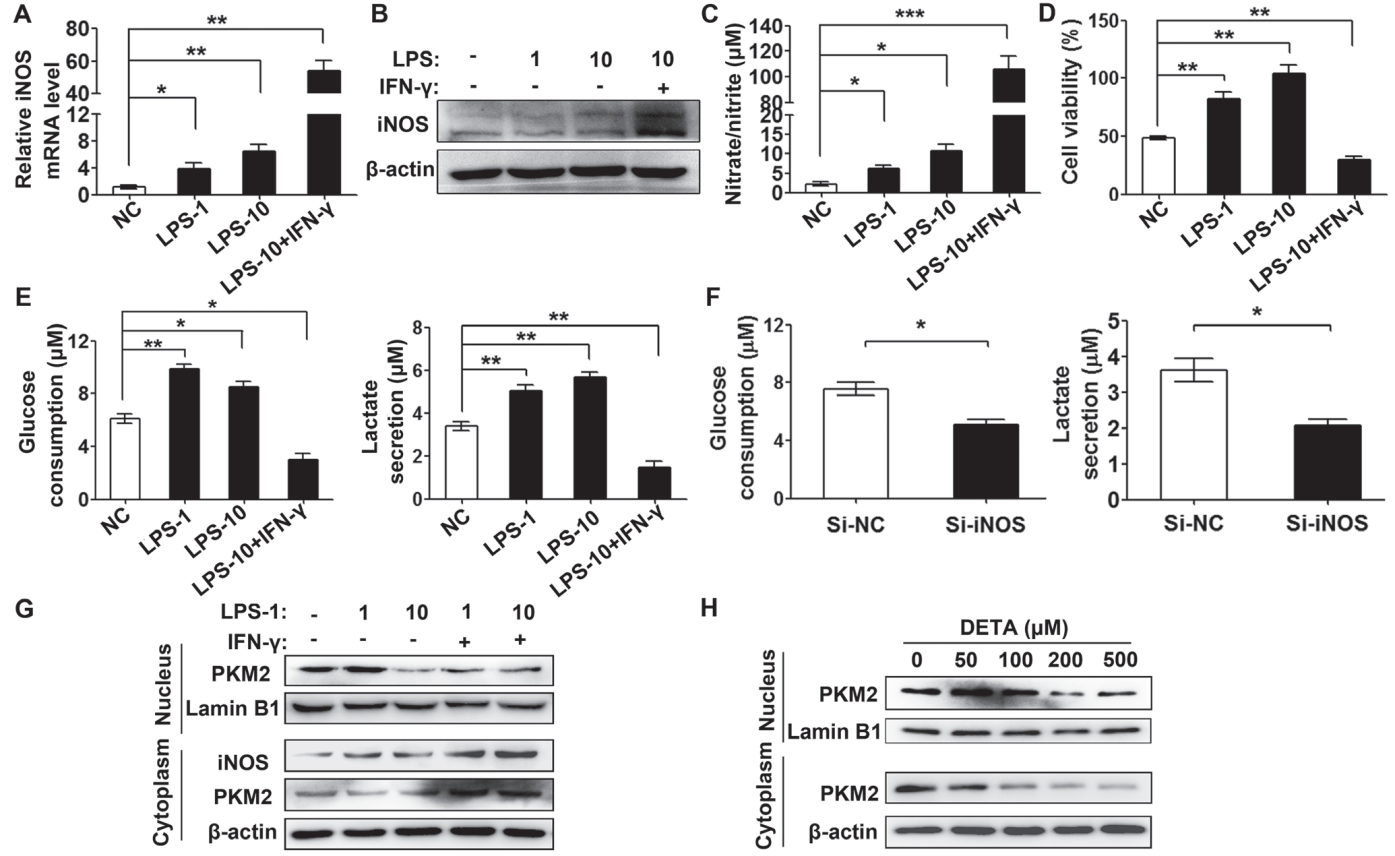
Dual role of iNOS in ovarian cancer glycolysis (**A**) Inflammatory stimuli increased iNOS mRNA expression as analyzed by real-time PCR. The SKOV3 cells were treated with LPS (1 μg/ml, 10 μg/ml) or LPS (10 μg/ml) plus IFN-γ (20 ng/ml) for 6 hours. (**B**) Inflammatory stimuli increased iNOS protein expression as analyzed by immunoblotting. The SKOV3 cells were treated as mentioned above for 24 hours. (**C**) Inflammatory stimuli increased nitric oxide production as determined by Griess assay. The SKOV3 cells were treated as mentioned above for 48 hours. (**D**, **E)**. Cell viability and glycolysis were increased by moderate inflammatory stimuli and attenuated by excessive inflammatory stimuli. SKOV3 cells treated as mentioned above for 48 hours were sent to CCK8 (D), glucose consumption (E, left panel) and lactate secretion (E, right panel) assay. (**F**) iNOS knockdown attenuated glucose consumption and lactate secretion in SKOV3 cells after transfected with si-iNOS or si-NC for 48 hours. (**G**) Dual effect of iNOS on PKM2 nuclear translocation as tested by immunoblotting. The SKOV3 cells were treated with LPS (1 μg/ml, 10 μg/ml) or LPS plus IFN-γ (20 ng/ml) for 48 hours. (**H**) Dual effect of nitric oxide on PKM2 nuclear accumulation. SKOV3 cells treated with different concentrations of DETA-NONOate were subjected to immunoblotting analysis. Columns, mean (*n* = 3); bars, s.e.m; **P < *P < 0.05; **P < 0.01; ***P < 0.001 in A and C–F.

To elucidate whether iNOS regulates glycolysis and cell proliferation in ovarian cancer, we stimulated iNOS expression by lipopolysaccharide (LPS) and interferon γ (IFN-γ) [34]. After treating with LPS alone (1 μg/ml and 10 μg/ml) or the combination of LPS (10 μg/ml) and IFN-γ (20 ng/ml) for 6 hours, the SKOV3 cells displayed 3 to 6-fold increased iNOS mRNA expression by stimulating with LPS alone and dramatically 50-fold by treatment of combining LPS with IFN-γ (Figure 6A). The iNOS protein level was slightly increased by LPS treatment alone but dramatically increased by combining stimulation of LPS and IFN-γ for 24 hours (Figure 6B). A Griess assay was performed to assess intracellular nitric oxide production. LPS alone increased nitrite production by 3 to 5 times (equally to NO of 30–50 nM) and LPS plus IFN-γ increased 50 times (equally to NO of 300-500 nM) in the culture media of ovarian cancer cells (Figure 6C).

Next we examined the effect of LPS and IFN-γ-induced iNOS induced on glucose consumption and lactate secretion in ovarian cancer cells. LPS alone stimulation increased glucose consumption and lactate secretion, whereas LPS plus IFN-γ significantly inhibited it (Figure 6E). To further confirm the role of iNOS in glycolysis, we knockdown of iNOS with siRNA in SKOV3 cells and found that iNOS knockdown reduced the glucose consumption and lactate secretion (Figure 6F and Supplementary Figure 2B). Consistently, alone LPS treatment enhanced cells proliferation, whereas treatment with LPS and IFN-γ attenuated it (Figure 6D). This is consistent with the above results, low/physiological nitric oxide to promote glycolysis and cell proliferation, while the excess nitric oxide inhibition of it.

To explore the correlation of iNOS regulation on glycolysis and PKM2 subcellular localization, we analyzed PKM2 in the nuclear and cytoplasmic fraction of SKOV3 cells stimulated by LPS and IFN. Low-dose LPS (1μg/ml) treatment increased PKM2 in the nuclear fraction, while PKM2 in the nuclear fraction was reversed in case of high-dose LPS (10 μg/ml) or LPS combined with IFNγ. Interestingly, the cytoplasmic PKM2 was also increased under the combined treatment of LPS and IFN-γ (Figure 6G). Collectively, these results indicated that moderate levels of iNOS are beneficial for tumor glycolysis and proliferation by producing nitric oxide at levels slightly above physiological condition (below 100 nM); and excess iNOS, which may be present in pathological conditions such as acute inflammation and reactive oxygen stress (ROS), is detrimental to glycolysis and leads to cytotoxicity of ovarian cancer cells.

### iNOS expression predicts an aggressive phenotype of ovarian cancer

**Figure 7:**
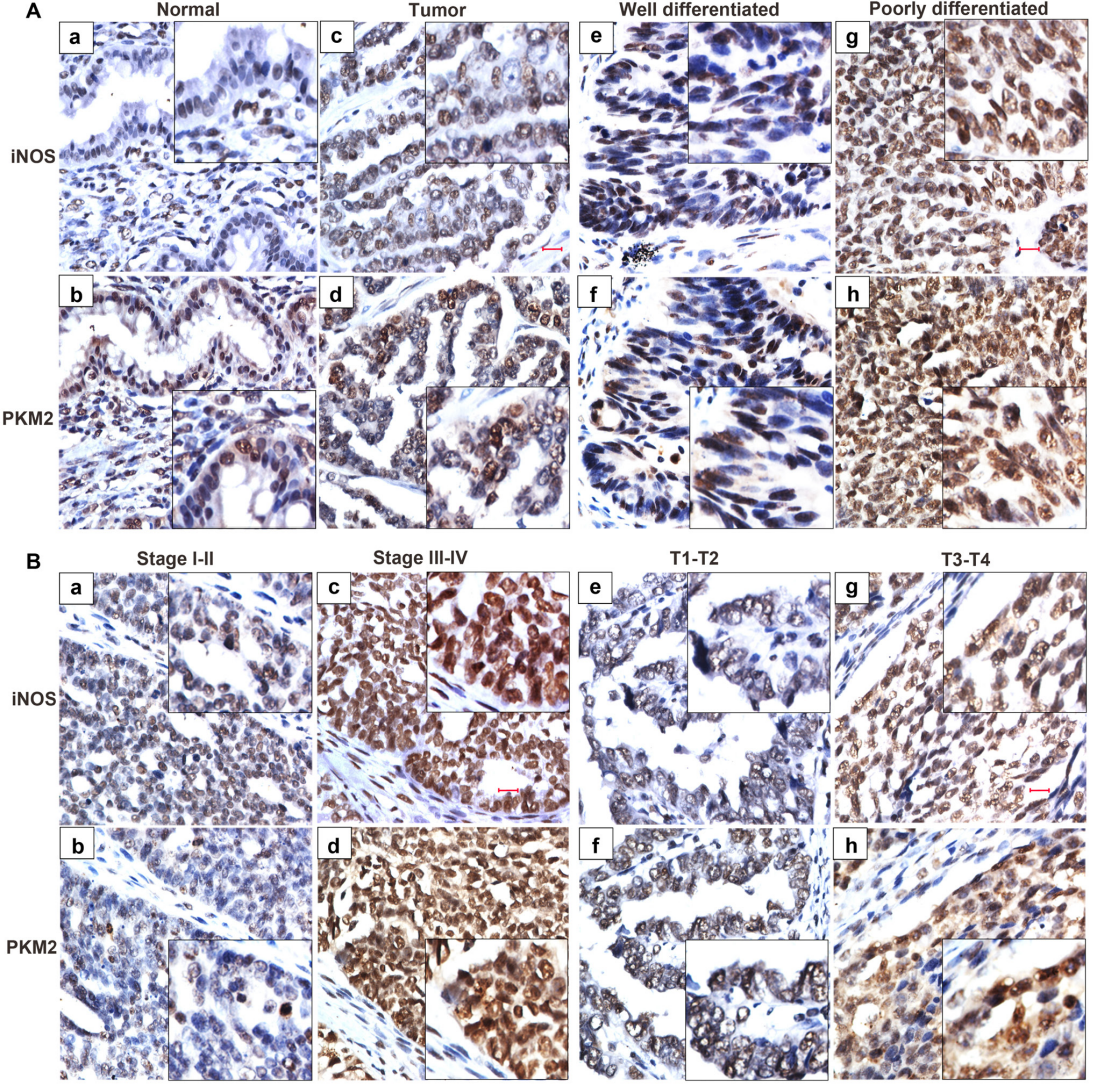
iNOS expression predicts an aggressive phenotype of ovarian cancer specimens The expression of iNOS and PKM2 is evaluated in 150 ovarian cancer tissues and 10 normal ovarian epithelial tissues by immunohistochemistry method. (**A**) iNOS and PKM2 staining in ovarian cancer tissues (c, d) is stronger than that in the normal ovarian epithelial tissues (a, b). The expression of iNOS and PKM2 were strongly stained in the poorly differentiated (g, h), while weakly stained in well differentiated ovarian cancer (e, f). (**B**) Representative images of iNOS and PKM2 in ovarian cancer tissues of different TNM stages. The expression of iNOS and PKM2 were strongly stained in the stage III-IV (c, d) and T3-T4 (g, h), while weakly stained in stage I-II (a, b) and T1-T2 (e, f) of cancer tissues.

To verify the clinical significance of iNOS/NO signaling and its correlation with PKM2 expression, we analyzed the expression of iNOS and PKM2 in 150 ovarian carcinomas and 10 normal ovarian epithelial tissues by immunohistochemistry assay. iNOS is present in the nucleus of both tumor epithelium and tumor-infiltrating cells, and tumor epithelial cells are likely to be the major source of iNOS in ovarian tumors (Figure 7A–7E). The degree of iNOS and PKM2 staining in ovarian malignant specimens was higher than that in non-cancerous ovarian epithelial biopsies (Figure 7A-a, b, c, d).

The correlation of iNOS expression with various clinicopathological characteristics of ovarian cancer patients was summarized in Table 1. The expression of iNOS was negatively correlated with the pathological grade (*P* = 0.001), race (*P* = 0.002), clinical stage (*P* = 0.047), and tumor size (T classification) (*P* = 0.037) in 150 ovarian tumor patients. There was no significant association between iNOS expression and age (*P* = 0.496), lymph node invasion (N classification) (*P* = 0.16), and metastasis (M classification) (*P* = 0.334). Briefly, iNOS overexpression was observed more frequently in poorly differentiated (Figure 7A–7G), high clinical staged (stage III–IV, Figure 7B–c), highly growth (T3–T4, Figure 7B–7G) and serous carcinoma cancer tissues. Additionally, iNOS was significantly associated with increased PKM2 expression (Figure 7A, 7B-b, d, f, h). These results suggest that high iNOS expression promotes aggressive phenotypes of ovarian tumors and PKM2 may be involved in the process.

**Table 1:** Association between the clinicopathological features and iNOS expression in 150 ovarian cancer patients

Patients					
Variables	*n*	iNOS staining (%)		X^2^	*p*
		Low (68)	High (82)		
Age (y)					
< 48	54	28 (52)	26 (48)	0.691	0.496
≥ 48	96	43 (45)	53 (55)		
Grade					
3–4	50	14 (28)	36 (72)	11.245	0.001
1–2	100	57 (57)	43 (43)		
Histological subtype					
Serous carcinoma	118	48 (41)	70 (59)	9.828	0.002
Non-serouscarcinoma	32	23 (72)	9 (28)		
Tumor stage					
I–II	86	47 (55)	39 (45)	4.33	0.047
III–V	64	24 (38)	40 (63)		
T classification					
T1–T2	101	54 (54)	47 (47)	4.664	0.037
T3–T4	49	17 (35)	32 (65)		
Lymph node metastasis					
Yes	47	18 (38)	29 (62)	2.242	0.16
No	103	53 (52)	50 (49)		
Distant Metastasis					
Yes	10	3 (30)	7 (70)	1.291	0.334
No	140	68 (49)	72 (51)		
PKM2 expression					
Low	48	35 (73)	13 (27)	18.533	0.000
High	102	36 (35)	66 (65)		

T: tumor size; N: lymph node metastasis; M: distant metastasis.

## DISCUSSION

The role of nitric oxide in tumor biology is quite complex and perplexing, and both promotion and inhibition of tumor growth have been described [6]. It has been reported that nitric oxide promotes glycolysis in ovary cancer cells, but the mechanism was unknown [11]. In this study, we elucidated the dual effects of exogenous nitric oxide on glycolytic regulation: at low concentration, nitric oxide promotes glycolysis and inhibits at high concentration. EGFR/ERK2-dependent phosphorylation and nuclear translocation of PKM2 is involved in the nitric oxide-induced glycolysis. We further found that LPS-induced iNOS enhanced glycolysis in ovarian cancer cells and iNOS mRNA levels predict poor prognosis in patients with ovarian cancer. Moreover, the correlation between iNOS and PKM2 expression may imply that iNOS regulates glycolysis in cancer cells by modulating PKM2.

The dual effect of nitric oxide on glycolysis is concentration-dependent: low levels of nitric oxide promotes the metabolic shift to glycolysis for unlimited cell proliferation and oxidative defense [12], while excess nitric oxide causes mitochondrial damage and attenuated glycolytic enzymes, leading to glycolysis inhibition and tumor cytotoxicity [35, 36]. Nitric oxide is catalyzed by three NOS isoforms with different express patterns and activities. nNOS and eNOS produce constitutively low levels of NO, which usually promotes tumor growth in many cellular processes such as cell proliferation, anti-apoptosis and migration [37]. However, the effect of iNOS on cancer progression was controversial [38]. This study showed that LPS-induced low levels of iNOS promote glycolysis to produce biomass, ATP and NADPH for cancer cell proliferation requirements. The combination of LPS and IFN-γ-induced high levels of iNOS yielded excess of nitric oxide that inhibits glycolysis, which may provide a good explanation for the inhibitory effect of iNOS on tumors under inflammatory conditions.

PKM2 exists as inactive monomer, low active dimer and high active tetramer in cells [18]. In response to various growth stimuli, PKM2 is converted from a high active tetramer to a low active dimer, resulting in the accumulation of glycolytic intermediates for macromolecule biosynthesis [21]. In addition, the dimer PKM2 can be translocated into the nucleus and act as a transcriptional coactivator of HIF-1α and c-MYC to regulate glycolytic gene expression [18]. Here we established a link between NO/iNOS signaling and PKM2 nuclear translocation: low/intermediate concentrations of nitric oxide promote glycolysis and PKM2 nuclear translocation in EGFR/ERK2-dependent manner. Actually, this phenomenon is not limited in ovary cancer cells, but also in breast cancer and colon cancer (unpublished data). It was reported that low levels of nitric oxide can induce ERK phosphorylation in a sGC-dependent manner [39]. This study shows that nitric oxide-regulated glycolysis seems to be partially mediated by the sGC-cGMP signaling pathway, which extends our knowledge on the nitric oxide-regulated glycolsis by activating multiple signaling pathways. Additionally, our results showed that exogenous nitric oxide had little effect on the S-nitrosylation of PKM2, while the pan NOS inhibitor L-NAME reduced it, suggesting that endogenous nitric oxide formed by NOS isoforms may cause S-nitrosylation of PKM2.

**Figure 8:**
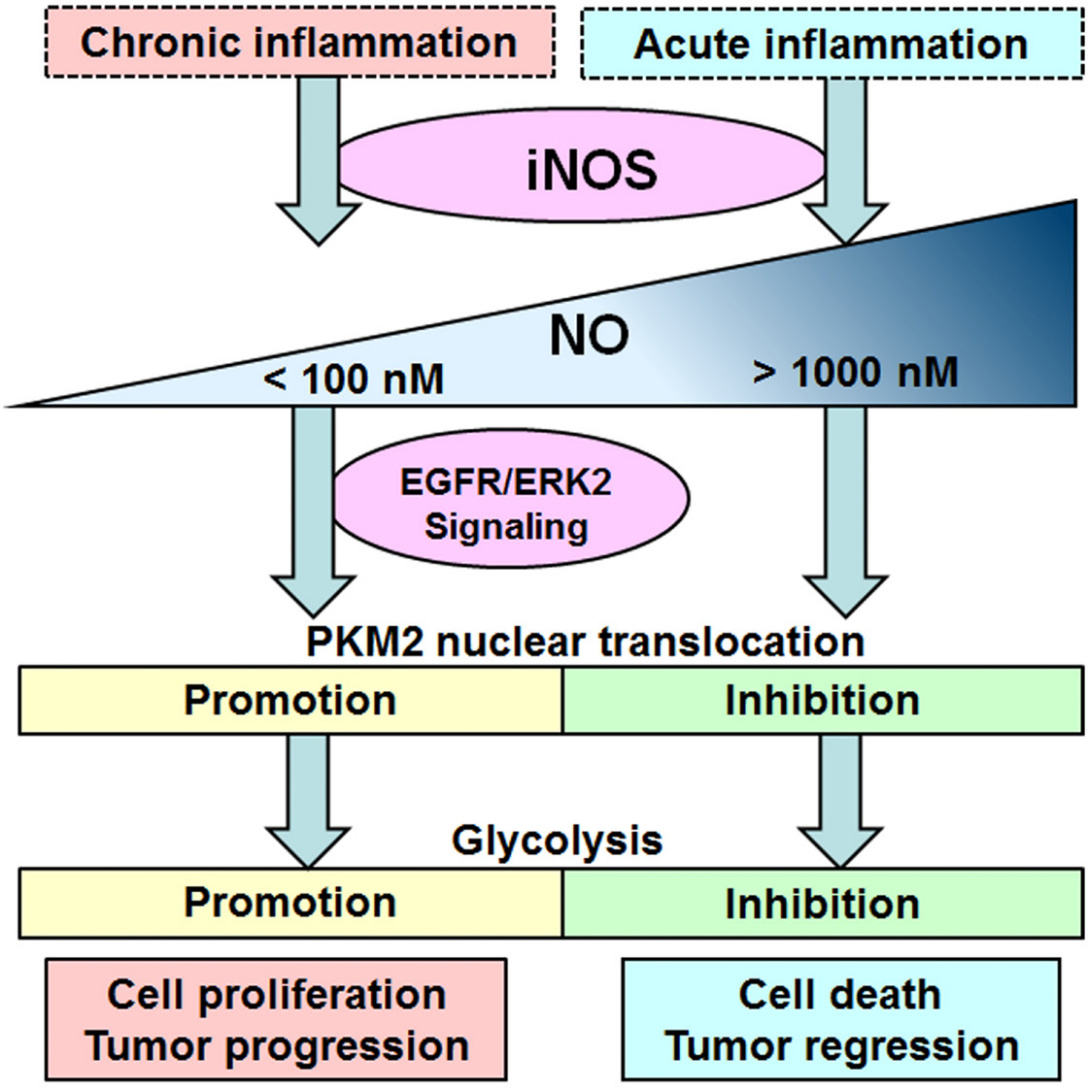
Schematic representation the dual role of iNOS/NO in cell glycolysis Chronic inflammation stimulates mild iNOS expression to produce nitric oxide slightly above physiological condition, which contributes to glycolysis and cell proliferation through inducing the PKM2 nuclear translocation in EGFR/ERK2-dependent manner. Acute inflammation stimulates iNOS expression to produce excess nitric oxide, which inhibits the nuclear translocation of PKM2 resulting in the glycolysis inhibition and tumor death.

Tumor-associated inflammation is now identified as one of hallmarks of cancer [40]. Inflammation promotes tumorigenesis and progression by supplying bioactive molecules to tumor microenvironment, including cytokines, chemokines, growth factors, reactive oxygen and nitrogen species, to sustain proliferative signaling and facilitate angiogenesis, invasion and metastasis [41]. iNOS expression and its nitrotyrosine production in inflammation are involved in tumorigenesis and progression [42]. In addition, the expression of iNOS in macrophages is less in chronic inflammatory and tumor tissues than in acute inflammatory tissues [43]. In this study, iNOS promotes tumor glycolysis and proliferation under mild inflammatory stimulation, which suggests that iNOS expression in tumor-associated macrophages may release low levels of nitric oxide in the tumor microenvironment to promote tumor glycolysis and progression (Figure 8).

Increased iNOS expression is positively correlated with malignancy in gastric cancer, hepatocellular carcinoma, melanoma and leukemia [37, 38], and negative correlation was also observed in other cancers such as lung cancer [44]. Our study found that iNOS expression is positively correlated with PKM2 and other glycolysis enzymes. Over-expressing iNOS predicts aggressive clinicopathologic characteristics and poor survival outcome of ovarian cancer patients, supporting the idea of iNOS expression as a useful prognostic marker for patients with ovary cancer. Recent studies have been devoted to developing anti-tumor therapies targeting tumor metabolism [45]. To date, both anti-NO and NO-based therapeutic approaches are under development [46]. Our study suggests that iNOS inhibitors in combination with glucose derivatives may provide an effective approach for inhibition of tumor growth. The level of endogenous or artificially induced nitric oxide in the tumor microenvironment must be taken into consideration in designing a nitric oxide-based therapeutic strategy against cancer.

## MATERIALS AND METHODS

### Cell lines and reagents

The human ovarian cancer cell lines SKOV3, OVCAR3 and ES-2 were purchased from the American Type Culture Collection (ATCC) and were routinely tested for absence of mycoplasma using a Mycoplasma Detection Kit (Bitool, China). All the cells were grown in Roswell Park Memorial Institute (RPMI)-1640 medium (Gibco, Gaithersburg, MD, USA) supplemented with 10% fetal bovine serum (Biological Industries, Beit Haemek, Israel) and maintained at 37°C in a humidified atmosphere of 5% CO2. The chemicals DETA-NONOate and L-NAME obtained from Cayman Chemical were dissolved in ddH_2_O and DMSO respectively. Glucose analog 2-deoxy-D-glucose (2-DG, dissolved in distilled water, #D8375), soluble guanylyl cyclase inhibitor 1H-[1,2,4] oxadiazolo [4,3-a]quinoxalin-1-one (ODQ, dissolved in DMSO, #O3636), EGF receptor (EGFR) inhibitor Tyrphostin AG 1478 (dissolved in DMSO, #T4182) and MEK/ERK inhibitor U0126 monoethanolate (dissolved in DMSO, #U120) were purchased from Sigma-Aldrich (St Louis, MO).

### Nitrate/nitrite measurement

The quantification of nitric oxide was based on nitrate/nitrite production by fluorometric method. Cells were seeded in a 6-well plate at 40–50% confluence and co-cultured with L-NAME (1 mM) or DETA-NONOate (50 μM) for 24 hours. The Nitrate/Nitrite Fluorometric Assay Kit (#780051, Cayman Chemicals, Ann Arbor, MI, USA) was used according to the manufacturer's instructions. Briefly, 10 μl supernatant of each sample was mixed with 70 μl assay buffer in a 96-well plate, followed by adding enzyme cofactor and nitrate reductase mixture. After incubation for 30 minutes, DAN Reagent and NaOH were added into each well. The plate was immediately read using a fluorometer at the excitation wavelength of 365 nm and emission wavelength of 430 nm.

### Cell viability assay

Ovarian cancer cells were seeded in 96-well culture plates at a density of 2,000–3,000 cells per well and treated with indicated chemicals (L-NAME, DETA-NONOate or 2-DG) for 48 hours. Cell counting kit reagent (#BB-4202, BestBio, Shanghai, China) was added to each well and incubated for 1–3 hours. The absorbance was read at 490 nm using a spectrophotometer (BioTek, Winooski, VT, USA). Cell ability was expressed as absorbance relative to that of untreated controls.

### Colony formation assay

Cells were plated into 6-well plates at a density of 500 cells per well. 24 hours later, cells were treated with different chemicals and incubated in complete culture medium for 14 days. The cells colonies were fixed with 10% formaldehyde (Roche) and stained with crystal violet solution for 30 min at room temperature. The plates were scanned and the number of colonies with > 50 cells was calculated by the Image J software (NIH, Bethesda, MD, USA).

### Apoptosis assay

Annexin V-FITC Apoptosis Detection Kit (keygen Biotech) was used according to the manufacturer's protocol. Cells were grown in 6-well plates and treated with different chemicals. After 48-hour incubation, cells were harvest, washed with cold PBS, and re-suspended in binding buffer (1 × 10^7^cell/mL). 5 μl Annexin V-FITC and 5 μl propidium iodide were added into each sample. After incubating for 15 min in dark place at room temperature, samples were analyzed using a FACSAria Flow cytometer (Beckton Dickson, San Jose, CA, USA).

### Nude mice and tumor xenograft experiments

Animal experiments were approved by the Ethical Committee for Animal Research of Southern Medical University (protocol number: 2011–020) and conducted in strict accordance with the guidelines from the Ministry of Science and Technology of China. BALB/c-nu female mice (4–6 weeks old, 18–20g) were purchased from Guangdong Medical Laboratory Animal Center and housed in SPF (specific pathogen free) facilities on a 12-hours light/dark cycle. 12 mice were randomly assigned to two groups (*n* = 6/group) and inoculated with SKOV3 cells (5 × 10^6^ cells in 150 μl PBS) subcutaneously in the dorsal flank. On day 14, the two groups intraperitoneally injected with L-NAME (50mg/kg, diluted in 0.9% NaCl, text group) and 0.9% NaCl (control group) respectively twice a week for 35 days. On day 5 and day 30, two mice were died off with unknown reason, so the two mice were excluded from the experimental system. Tumors length (L) and width (W) were measured with vernier calipers weekly and tumor volumes were calculated using the formula V= (L × W^2^)/2. The animals were euthanized on day 50. The tumors tissues were excised and weighed, parts of the fresh tissues were paraffin-embedded for immunohistochemiscal analysis.

### Glucose consumption assay and lactate secretion assay

Cells were seeded in 6-well plates at a density of 5,000 cells per well and treated with DETA-NONOate or L-NAME for 24 hours, the culture media were collected. Glucose Colorimetric Assay Kit II (# K686-100, BioVision) was used for glucose consumption assay according to the manufacturer's instruction. Briefly, reaction mixture consists of 2 μl glucose substrate and 2 μl glucose enzyme was diluted in 46 μl glucose assay buffer and added into a 96-well plate. The test samples of 1 μl culture media diluted in 49 μl glucose assay buffer were also added into the plate and incubated at 37°C for 30 minutes in the dark. Absorbance at a wavelength of 450 nm was determined using a spectrophotometer.

Lactate Colorimetric Assay Kit II (# K627-100, BioVision) was used for lactate secretion assay according to the manufacturer's instruction. Briefly, reaction mixture consists of 2 μl lactate substrate and 2 μl lactate enzyme was diluted in 46 μl lactate assay buffer and added into a 96-well plate. The test samples of 1 μl culture media diluted in 49 μl lactate assay buffer were also added into the plate and incubated at 37°C for 30 minutes in the dark. Absorbance at a wavelength of 450 nm was determined using a spectrophotometer.

### ATP measurement

Cell Titer-Glo 2.0 Assay (Promega, Madison, WI, USA) was used to determine the amount of cellular ATP according to the manufacturer's protocol. Briefly, cells were seeded in 96-well culture plates (5,000 cells per well). After indicated treatment and 48-hour incubation, culture media was removed and Cell Titer-Glo regent 100 μl per well) was added. Plate was incubated in dark for 10 minutes and the luminescence was read by the Panomics Luminometer (Affymetrix, Santa Clara, CA, USA).

### NADPH measurement

NADPH assay was modified from the protocol by Swenberg JA's team [47]. Briefly, stock solutions of XTT (251 mM in DMSO) and 1-methoxy-5-methylphenazinium methylsulfate (PMS, 0.5 Mm in DMSO) were prepared. A PMS/XTT solution was then prepared by dissolving 3.2 ml XTT in 66.6 μl PMS. Cells seeded in 96-well plates at a density of 2000–4000 cells/well, and then send to transfection or chemical treatment as indicated. After 24 hours of incubation, media was removed, 100 μl XTT/PMS reagent was added to each well. Media containing XTT/PMS was added to the wells. Plates were incubated for 3 hours and send to read in a spectrophotometer at 450 nm with 650 nm as the reference filter.

### Small interfering RNA (siRNA) transfection

PKM2 (Gene ID: 5315) and iNOS (Gene ID: 4843) knockdown were achieved by transcriptional transfection of siRNA oligonucleotides approach. The PKM2 siRNA sequence was derived from Hu's report [48] and synthesized by Invitrogen (Shanghai, China); the iNOS siRNA sequence was designed and synthesized by RiboBio (Identification number: siB1117165444; Guangzhou, China). The target sequences for siRNAs are listed below. PKM2 siRNA: 5′-C CAUAAUCGUCCUCACCAATT-3′, iNOS siRNA: 5′- AATATTACGGCTCCTTCAAAG-3′, negative control (NC) siRNA: 5′-UUCUCCGAACGUGUCACGUTT-3′. The day before transfection, ovarian cancer cells were seeded at a density of 5 × 10^5^ cells per well in 6-well plates and cultured without penicillin and streptomycin overnight. OPTI-MEM serum-free medium (Gibco, USA) and lipofectamine^™^ 2000 (Invitrogen, Waltham, USA) were used for transient transfection experiments. The siRNAs with a final concentration of 100 nM and 4 μl lipofectamine 2000 was added to each well. The transfection efficiency was verified by immunoblotting.

### Western blotting analysis

RIPA lysis buffer supplemented with the PMSF and phosphatase inhibitor cocktail (Beyotime, China) was used for total protein extraction. Nuclear and Cytoplasmic Protein Extraction Kit (#FD0199, FUDE Biology) was used for harvesting nuclear and cytoplasmic protein according to the manufacturer's instructions. Protein concentration was determined with BCA protein assay kit (# E162-01, KeyGen Biotech). Equal amounts of protein were separated by SDS-PAGE and transferred to a PVDF membrane (Millipore, Billerica, USA). The membrane was blocked with 5% bovine serum albumin and probed with the appropriate primary antibodies: rabbit anti-PKM2 (#ab150377, 1: 1000 dilution) and rabbit anti-lamin B1 (#ab133741, 1: 1000 dilution) were from Abcam (Cambridge, UK); rabbit anti-p-AKT1/2 (1: 500 dilution), rabbit anti-EGFR (1: 500 dilution), rabbit anti-non phosphor (active) β-catenin (#8814, 1: 500 dilution) and rabbit anti-p-ERK (42/44) (1: 500 dilution) were from Cell signaling technology (Danvers, MA), rabbit anti-PKM2(phosphor Ser37) (#GTX54549, 1: 500 dilution, GeneTex, USA), or mouse anti-p-STAT3 (s727) (sc293059, 1: 500 dilution, Santa Cruz Technology, California). After incubating overnight at 4°C, the membrane was then probed with horseradish peroxidase (HRP) conjugated goat anti-rabbit (or goat anti-mouse) IgG secondary antibody (Proteintech, USA). Signals were visualized using the eECL Western Blot Kit (#P90720, Millipore).

### Immunofluorescence assay

Cells were seeded on coverslips and treated with DETA-NONOate (1 mM) for 24 hours. Cells were fixed with 4% paraformaldehyde for 30 minutes and permeabilized by 0.1 % Triton X-100. After blocking with 5% BSA, cells were probed with the primary rabbit anti-PKM2 monoclonal antibody (1: 400, Abcam) overnight at 4°C and the corresponding Alexa Fluor 488 goat anti rabbit IgG secondary antibody (#ZF-0511, ZSGB-BIO, China). The coverslips then counter-stained and mounted with DAPI (Sigma-Aldrich) mixed with anti-fade fluorescence mountants (Life technologies) at the ratio of 1: 1. Analysis was performed using a florescence microscope (Nikon Eclipse Ti-U, Japan).

### Measurement of pyruvate kinase activity

The pyruvate kinase activity for cell lysates was determined by a lactate dehydrogenase coupled assay using Pyruvate Kinase Detection Kit (#A106, Nanjing jiancheng bioengineering institute, China). Briefly, the indicated reagents and equal amount of proteins were mixed and the initial absorbance was read at 340 nm (recorded as A1) by a spectrophotometer. Following incubation at 37°C for 15 minutes, the absorbance at 340 nm was changed due to the oxidation of NADH and recorded as A2. One unit of catalytic activity induces the oxidation of 1 μmol NADH per minute at 37°C and pH 7.6. The formula used for the detection of pyruvate kinase activity was: The pyruvate acitivity (U/gprot) =

$$\frac{A1-A2}{\text{Mec}\left(6.22\right)}÷\text{RT}\left(15\;\min\right)÷\text{Cdop}\left(0.5\text{ cm}\right)\times \frac{\text{Total volume}\left(1.195\right)}{\text{Sample volume}\left(0.02\right)}÷\text{Sample Concentration}\left(\text{gprpt}/\text{ml}\right)$$

(A1: Initial absorbance at 340 mm; A2: Reactived absorbance at 340 mm; Mec: Millimoles extinction coefficient; RT: reaction time; Cdop: colorimetric dish optical path)

### Quantitative real-time PCR (qRT-PCR)

Total RNAs were extracted from ovarian cancer cells using Trizol reagent (Invitrogen, USA). Reverse transcription reaction with random primers was done using PrimeScript RT reagent Kit (TaKaRa, Japan). Quantitative real-time PCR was performed using SYBR Premix ExTaq (TaKaRa, Japan) on the Mx3005P Real-Time PCR System (Stratagene, La Jolla, CA). The reactions were initiated with a denaturing step at 95°C for 2 minutes, followed by 40 cycles at 95°C for 30 seconds. The primers were listed in Supplementary Table 1. β-actin was used as endogenous control. All samples were normalized to endogenous control and the relative quantification of mRNA was determined by the 2^−∆∆Ct^ method. The experiments were done at least thrice independently and all samples were in triplicate.

### Immunohistochemistry (IHC)

The paraffin-embedded tumor tissues from mice were cut into 4 μm sections. The ovarian histopathology was confirmed by eosin (H&E) staining and then sent to immunohistochemistry staining. Following deparaffinization and rehydration, the paraffin- embedded sections were subjected to high pressure for 3 min for antigenic retrieval. The slides were incubated overnight at 4°C with the following primary antibodies: mouse anti-iNOS (1: 200 dilution, AF0199, Affinity), rabbit anti-PKM2 (1: 200), rabbit anti-p-ERK1/2 (1: 50) or rabbit anti-EGFR (1: 100). The sections were then incubated with second antibody and stained with the 3.3-diaminobenzidine (DAB) solution (ZSGB-Bio, Beijng, China). Nuclei were counterstained with hematoxylin. The staining intensity of tumor cells was scored as follows: 0 - no staining, 1 - weak staining; 2 - modest staining and 3 - strong staining. The positive staining ratio of tumor cells was classified into four grades as follows: 0 - no positive tumor cells, 1 - < 10% positive tumor cells, 2–10–50% positive tumor cells and 3 - > 50% positive tumor cells. The general IHC results were calculated by multiplying the positive staining grade with the intensity grade (0, 1, 2, 3, 4, 6, and 9). Finally, general IHC results ≤ 4 and ≥ 6 were defined as low and high expression respectively. Two pathologists examined and scored IHC results blindly without knowing the clinical characteristics and prognosis.

### S-nitrosylation detection assay

S-nitrosylated Protein Detection Assay Kit (Cayman, USA) based on the “Biotin-switch” method [49] was used for S-nitrosothiol detection according to the manufacture's instruction. All steps were done with minimal light exposure. Briefly, 100–250 μg protein lysates were extracted from SKOV3 cells treated with DETA-NONOate (50 μM) or L-NAME (1 mM). PKM2 protein was purified by immunoprecipitation (TR0064.0, ThermoFisher Scientific, Waltham, MA) and its free thiols were blocked by blocking agent. S-nitrosothiols in the protein were reduced to free thiol(s) and subsequently covalently labeled with maleimide-biotin. After concentration measurement and dilution with SDS loading buffer, the labeled proteins were denatured at 95°C. Samples were sent to immunoblotting with HRP-detected reagent and visualized by the eECL Western Blot Kit (#P90720, Millipore).

### Tissue microarray analysis

The expression of iNOS in human ovarian cancers was analyzed in two tissue microarrays (OV803a and OV809, US Biomax, Rockville, Maryland, USA) containing 150 ovarian cancer tissues and 10 non-cancerous ovarian epithelial tissues. The mean age was 48.1 ± 12.1 years, 42 cases (28.0%) were in grade 1, 58 cases (38.7%) were in grade 2, 50 cases (33.3%) were in grade 3. 58 cases (38.7%) were in stage I, 28 cases (18.7%) were in stage II, 54 cases (36%) were in stage III, 10 cases (6.7%) were in stage IV. Lymph node metastasis was present in 47 (31.3 %) patients, absent in 101 (67.3%) patients and not assessed in 2 (1.4%) patients. Distant metastasis was present in 10 (6.7%) patients, absent in 101 (93.3%) patients. Evaluation of histological tumor type showed that, 66 cases (44%) were serous adenocarcinoma, 52 cases (34.7%) were serous papillary adenocarcinoma, 16 cases (10.7%) were mucinous adenocarcinoma, 2 cases (1.3%) were mucinous cystadenocarcinoma, 2 cases (1.3%) were mucinous papillary adenocarcinoma, 6 cases (4%) were endometriosis adenocarcinoma, 2 cases (1.3%) were transitional cell carcinoma, 4 cases (2.6%) were clear cell carcinoma. Each TMA core was independently scored for iNOS and PKM2 IHC intensity.

### Statistical analysis

Statistical analyses were completed using SPSS ver. 19.0 software (IBM SPSS Inc., Chicago, IL, USA). All values are expressed as mean ± standard error. Two-tailed Student's *t-test* was used for comparison of two independent groups, one-way ANOVA analysis was made to compare difference between multiple groups, spearman's correlation coefficient was used to determine the expression correlations between iNOS mRNA and glycolytic genes. Kaplan-Meier method was used to evaluate the overall survival rate of ovarian cancer patients. Chi-square test was used to analyze the correlation between clinicopathological characteristics and iNOS expression. *P* values < 0.05 were considered significant. All cellular experiments were repeated at least three times.

## SUPPLEMENTARY MATERIALS FIGURES AND TABLES


