# Supplementary material for: iNOS-derived nitric oxide promotes glycolysis by inducing pyruvate kinase M2 nuclear translocation in ovarian cancer
Source: Oncotarget. 2017 Mar 23;8(20):33047–63. doi: 10.18632/oncotarget.16523 (PMC5464849; doi:10.18632/oncotarget.16523)
Supplement: Supplementary file 1 [file oncotarget-08-33047-s001.pdf]

iNOS-derived nitric oxide promotes glycolysis by inducing pyruvate kinase M2 nuclear translocation in ovarian cancer

Supplementary Materials

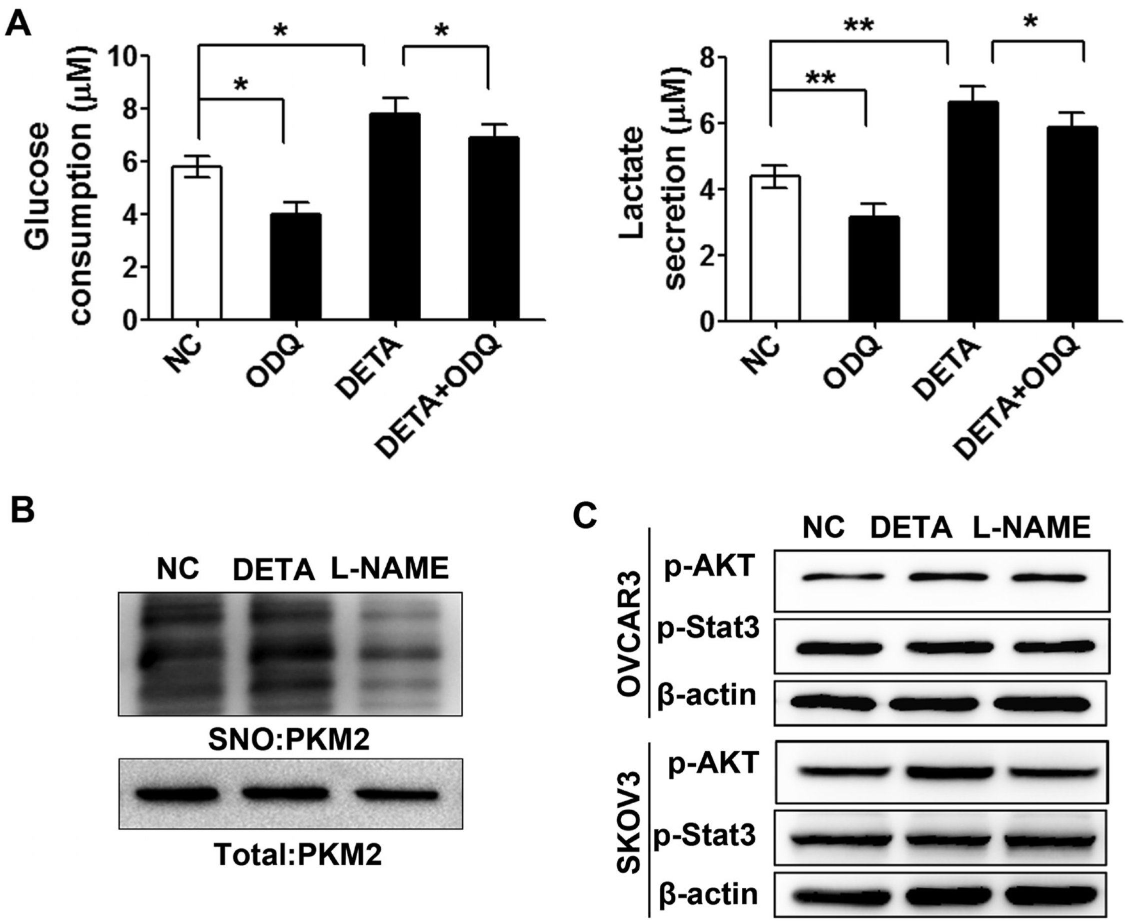

Supplementary Figure 1: (**A**) Nitric oxide-regulated glycolysis is partially mediated by sGC-cGMP signaling. Glucose consumption and lactate secrete were detected in SKOV3 cells after treated with DETA-NONOate, ODQ or their combination for 24 hours. Columns, mean (n = 3); bars, s.e.m; $^{**}P < 0.01$. (**B**) Biotin-switch assay was carried out to detect the PKM2 S-nitrosylation in SKOV3 cells after treated with DETA-NONOate and L-NAME for 24 hours. (**C**) Unchanged levels of phospho-STAT3 and phospho-AKT as detected by immunoblotting in OVCAR3 and SKOV3 treated with DETA-NONOate (50 μM) or L-NAME (1 mM) for 24 hours.

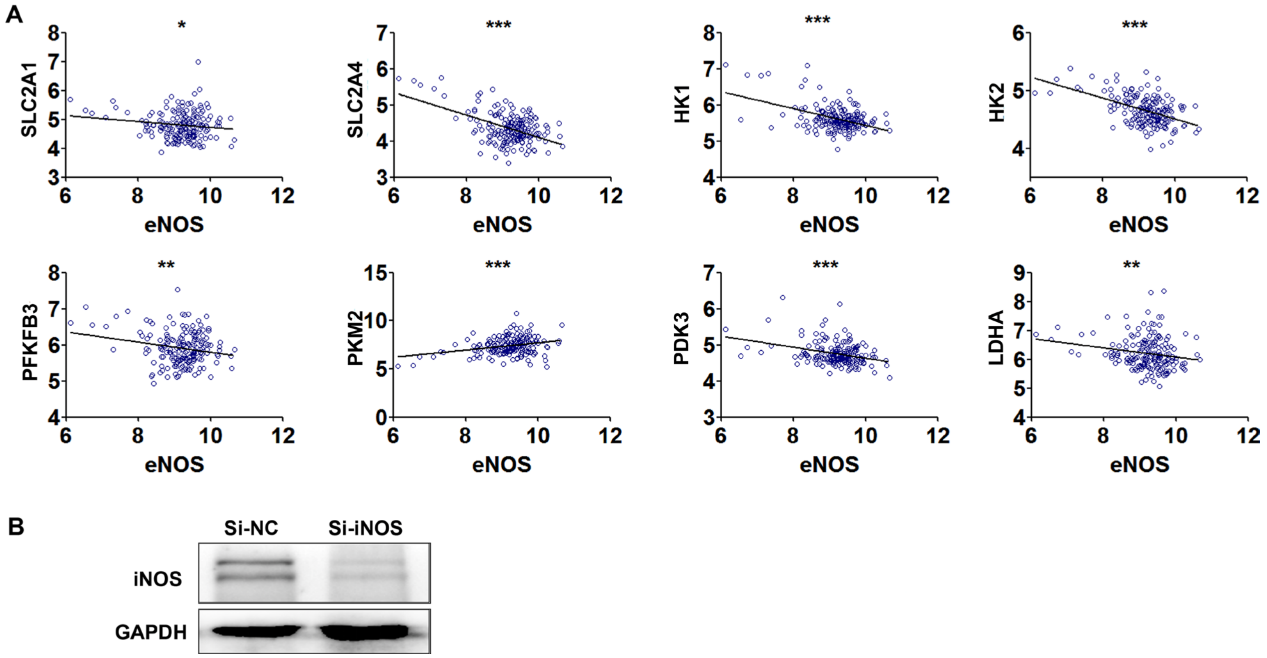

Supplementary Figure 2: (**A**) Scatter plot shows the negative correlation of eNOS mRNA values (x axis) with glycolytic genes *SLC2A1*, *SLC2A4*, *HK1*, *HK2*, *PFKFB3*, *PKM2*, *PDK3*, and *LDHA* (y axis) in 250 ovarian cancer patients. Global gene expression profiling of ovarian cancer was downloaded from Gene Expression Omnibus (GEO, GSE26712 and GSE14764).$^{*}P < 0.05$; $^{**}P < 0.01$; $^{***}P < 0.001$. (**B**) iNOS was successfully knocked down by si-iNOS in SKOV3 cells as tested by immunoblotting assay.

Supplementary Table 1: Primers for real-time PCR

Gene name	Forward (5′→3′)	Reverse (5′→3′)
GLUT1	CAGGAGATGAAGGAAGAG	TCGTGGAGTAAT AGA AGAC
GLUT3	CGGCTTCCTCATTACCTTC	GGCACGACT TAG ACATTGG
HK1	GGGTCCTGCTGGTCCGTGTT	TCCTCCCCTCGTCTCCTTCC
HK2	GGGTCCTGCTGGTCCGTGTT	TCCTGCGGGATGGCGTAGAT
PFK1	CCGCATCAAGCAGTCAGCAG	AGCCAGGTAGCCACAGTAGC
PDK2	TGAAGATGAGTGACCGAGG	GCAATCCATAACCAAAACC
LDHA	TGAAGTCGGCCTGATCATCG	GACACCAGCAACATTCATTCC
PKM2	TGACGAGAACATCCTGTGGC	GGAAGTCGGCACCTTTCTGC
iNOS	GAAGCGGAGACCCAAGAGA	TCGCAAAGAGGATGGTGACT
β-actin	GTGGCCATCTCTTGCTGCAAG	GGGAAATCGTGCGTGACATTAAG
